# Deciphering next-generation pharmacogenomics: an information technology perspective

**DOI:** 10.1098/rsob.140071

**Published:** 2014-07-16

**Authors:** George Potamias, Kleanthi Lakiotaki, Theodora Katsila, Ming Ta Michael Lee, Stavros Topouzis, David N. Cooper, George P. Patrinos

**Affiliations:** 1 Institute of Computer Science, Foundation for Research and Technology Hellas, Crete, Greece; 2 Department of Pharmacy, School of Health Sciences, University of Patras, University Campus, Rion, Patras, Greece; 3 Laboratory for International Alliance on Genomic Medicine, RIKEN Center for Integrative Medical Sciences, Yokohama, Japan; 4 Institute of Medical Genetics, School of Medicine, Cardiff University, Cardiff, UK

**Keywords:** whole-genome sequencing, personalized pharmacogenomics profile, informatics solutions, microattribution, drug metabolism, gene variants

## Abstract

In the post-genomic era, the rapid evolution of high-throughput genotyping technologies and the increased pace of production of genetic research data are continually prompting the development of appropriate informatics tools, systems and databases as we attempt to cope with the flood of incoming genetic information. Alongside new technologies that serve to enhance data connectivity, emerging information systems should contribute to the creation of a powerful knowledge environment for genotype-to-phenotype information in the context of translational medicine. In the area of pharmacogenomics and personalized medicine, it has become evident that database applications providing important information on the occurrence and consequences of gene variants involved in pharmacokinetics, pharmacodynamics, drug efficacy and drug toxicity will become an integral tool for researchers and medical practitioners alike. At the same time, two fundamental issues are inextricably linked to current developments, namely data sharing and data protection. Here, we discuss high-throughput and next-generation sequencing technology and its impact on pharmacogenomics research. In addition, we present advances and challenges in the field of pharmacogenomics information systems which have in turn triggered the development of an integrated electronic ‘pharmacogenomics assistant’. The system is designed to provide personalized drug recommendations based on linked genotype-to-phenotype pharmacogenomics data, as well as to support biomedical researchers in the identification of pharmacogenomics-related gene variants. The provisioned services are tuned in the framework of a single-access pharmacogenomics portal.

## 2. Introduction

A decade after the completion of the Human Genome Project, one would perhaps be forgiven for thinking that genomic medicine would be commonplace in clinical practice. However, it is clear that the growth of genomic knowledge has not led to a corresponding increase in clinical implementation. The concept of genomic or personalized medicine (GPM), the tailoring of medical treatment to the individual characteristics, needs and preferences of each patient, is not new. Indeed, it is pertinent to note the words of Hippocrates: ‘It is far more important to know what person the disease has than what disease the person has’. The discovery, made back in 1956, that the genetic basis for the selective toxicity of both fava beans and the antimalarial drug primaquine is a deficiency of the metabolic enzyme glucose-6-phosphate dehydrogenase [1] presents one of the earliest illustrations of the principle of personalized medicine.

Nowadays, it is almost axiomatic that the continuing advances in genomic research will revolutionize the way in which GPM is performed. Building on these advances, pharmacogenetics and pharmacogenomics, PGx^1^ in short, are currently driving discovery, analysis and interpretation in the context of research into the genetic basis of inter-individual variation in drug response [2]. As an integral part of GPM, PGx targets the delineation of the relationship between genomic variation/gene expression and drug efficacy and/or toxicity [3]. To date, there are several genes, referred to as pharmacogenes, which play a role in the absorption, distribution, metabolism, excretion and toxicity (ADMET) of several drugs. The most important ADMET genes can be grouped into four main categories: modifiers, phase-I and phase-II metabolism enzymes, and transporters. PharmaADME (http://www.pharmaadme.org/; an industry-initiated effort that aims to provide a core list of evidence-based drug metabolizing ADMET genetic biomarkers) catalogues 32 core (e.g. *ABCB1*—ATP-binding transporter), 267 extended (e.g. *AHR*—aryl hydrocarbon receptor), 73 related (not directly involved in metabolism, e.g. *CTSK*—cathepsin K) pharmacogenes, as well as 187 core genetic biomarkers (e.g. *CYP1A1*: c.2453C>A).

Moreover, in relation to oncology, the US National Cancer Institute (NCI) has announced a set of priorities that include treatment response and adverse outcomes associated with chemotherapeutic agents and other medications used to treat cancer (via the Trans-NCI Pharmacogenomics and Pharmacoepidemiology Working Group (PPWG); http://epi.grants.cancer.gov/pharm/ppwg.html). The recommendation for the corresponding research and development (R&D) agenda is directed towards: (i) supporting the routine collection of germline and tumour biospecimens from clinical trials and population-based studies; (ii) the development of, and support in, the identification of clinical, socio-demographic, lifestyle and genomic markers related to treatment response and/or adverse events; (iii) the incorporation of PGx markers into clinical trials; and (iv) addressing the ethical, legal, social, biospecimen, as well as data-sharing implications of PGx research [4].

The rise of next-generation sequencing technology has created unprecedented opportunities to analyse whole genomes [5]. This approach promises to be extremely useful in PGx, because unlike conventional medium- or even high-throughput genetic screening approaches, such as microarray-based assays (e.g. AmpliChip CYP450 (http://molecular.roche.com/assays/Pages/AmpliChipCYP450Test.aspx), Roche Molecular Diagnostics, Basel, Switzerland; and DMET Plus (www.affymetrix.com/estore/browse/level_three_category_and_children.jsp?category=35791&categoryIdClicked=35791&expand=true&parent=35923), Affymetrix, Santa Clara, CA, USA), it allows the acquisition of a full picture with respect to individual ADMET gene variants. This is important because it is very likely that each individual harbours rare and/or novel variants of functional significance in well-established pharmacogenes, which may render an individual/patient as a poor or hyper-metabolizer or non-responder to certain drugs, and which may go undetected when using a genetic screening assay.

**Figure 1.**
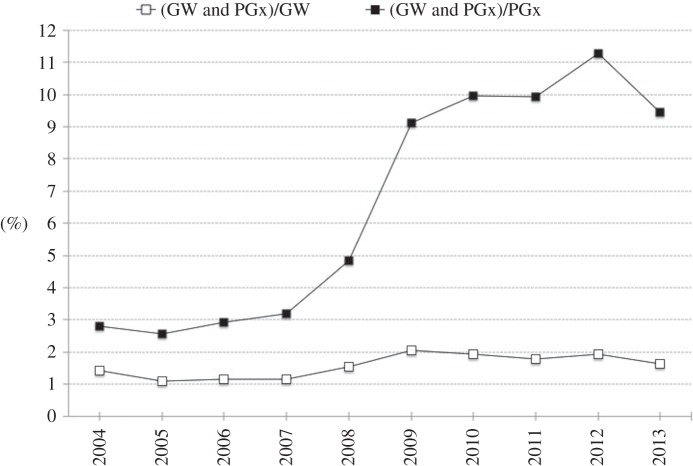
Publication over time of PubMed papers pertaining to search terms PGx (‘pharmacogenomics’ OR ‘pharmacogenetics’) and/or ‘genome-wide’ (GW). The dominance of the GW approach in PGx-related research (papers) has increased about fourfold over the last few years, from approximately 3% in 2004 to about 11% in 2012, with the dominance (over the same period) of PGx research in the GW research domain remaining about the same (around 1–2%). This demonstrates that the GW approach has drastically influenced PGx research.

Over the last few years, genome-wide association studies (GWAS) have been the main enablers of PGx research, with a track record of novel and interesting findings [6]. A search in PubMed (April 2014) for PGx-related papers shows that the proportion of papers related to ‘pharmacogenomics’ OR ‘pharmacogenetics’ (PGx) and ‘genome-wide’ (GW) as search terms has risen fourfold over the last 10 years, from about 3.0% in 2004 to more than 11% in 2012 (figure 1; fraction (PGx and GW)/PGx), with a fraction of about 2% for GW-related papers that involve pharmacogenomic quests (figure 1; fraction (PGx and GW)/GW)—similar results have been reported by Gurwitz & McLeod [7].

However, most variants that impact drug response still remain to be identified [8]. As GWAS may not identify all risk biomarkers, the identification of unknown (possibly rare) variants from whole-genome sequencing studies may provide indicative associations between specific genotypes and adverse drug reactions. Whole exome and/or whole-genome sequencing can now be easily performed using several commercially available or proprietary platforms, to analyse genome variation comprehensively and with a high degree of accuracy at reasonable cost, as compared with the recent past [9].

The paper is organized as follows: in §§2 and 3 we discuss recent advancements in PGx whole-genome sequencing, in §4 we highlight the current shortcomings of PGx data integration, while in subsequent sections (§§5–7) we describe the design principles and the development of an integrated PGx electronic assistant, as a potential approach to address the problem of PGx information management and overload, and the delivery of personalized PGx translation services.

## 3. Personalized PGx profiling using whole-genome sequencing

Recently, whole-genome sequence analysis of almost 500 individuals identified a very large number of rare potentially functional genomic variants in ADMET genes, which would not have been identified had a conventional high-throughput genetic screening approach been employed. Mizzi *et al*. [10] showed that the number of ADMET-related genomic variants identified by whole-genome sequencing was significantly higher compared with those that would have been identified had the DMET Plus assay, the most comprehensive genotyping platform for pharmacogenomic biomarkers available to date [11], been used. These authors reported the identification of 408 964 genomic variants in ADMET-related genes, of which almost 10% (38 636) attained population frequencies of more than 20%. On average, 17 733 variants were found for each individual of these 231 ADMET-related genes, compared with an anticipated 250 variants in the same genes had the DMET assay been employed. Interestingly, 16 487 novel (not annotated in dbSNP) variants were identified within exons and regulatory regions, of which 861 attained frequencies of over 1%, and are likely to be functionally significant. The latter finding underlines the fact that any result from a currently available pharmacogenetic screening assay would not be indicative of a patient's ability to respond to certain drugs and as such should be interpreted with a degree of caution.

In similar vein, Paré-Brunet *et al*. [12] resequenced an almost 700 kb DNA sequence including 23 vascular endothelial growth factor pathway genes that play a central role in physiology, pathophysiology and drug treatment in angiogenesis, and reported 3558 genetic variants of which 449 were novel. Similar applications of next-generation sequencing could also be envisaged for germline cancer variation discovery with possible PGx implementation to individualize cancer treatment [13].

It seems evident that, in the light of the plummeting cost of whole-genome sequencing and the gradual increase in data accuracy, one would envisage that comprehensive pharmacogenomic testing could be readily applicable in a clinical setting [14]. By applying whole-genome sequence analysis to two unrelated family members suffering from atrial fibrillation and presenting with differential response rates to anticoagulation treatment, Mizzi *et al*. [10] were able to delineate the differential response rate to anticoagulation treatment of these family members. In particular, whole-genome analysis in these family members not only revealed *CYP2C9* variants as the basis of the inter-individual response to acenocoumarol treatment, but was also able to predict the outcome of an alternative anticoagulation treatment using clopidogrel. Similar findings in genes involved in the metabolism of anti-cancer drugs [15] further demonstrate the potential applicability of this approach for pharmacogenomic testing in a clinical setting in the not too distant future.

## 4. Customized whole-pharmacogenome resequencing

**Figure 2.**
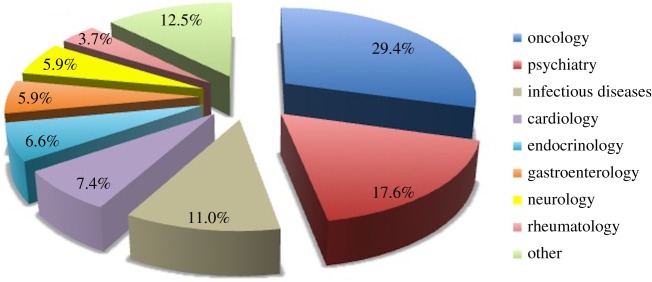
Disease distribution of PGx labelled FDA-approved drugs (‘other’ includes: haematology, dermatology, transplantation, urology, analgesia, anaesthesiology, antidotal therapy, autoimmune diseases, metabolic disorders and pulmonary diseases).

Although whole-genome sequence PGx analysis is still in its infancy, one might envisage that the ultimate pharmacogenomic test would involve at the very least the resequencing of the ADMET-related pharmacogenes, particularly those that have been acknowledged to be credible pharmacogenomic biomarkers by regulatory agencies. Several PGx tests have been developed, representing tangible deliverables from the numerous genomic studies that have attempted to correlate genetic variation with variable drug response. The US Food and Drug Administration (FDA) established the Genomics and Targeted Therapy Group (http://www.fda.gov/Drugs/ScienceResearch/ResearchAreas/Pharmacogenetics/ucm259430.htm) to advance the application of genomic technologies in the discovery, development, regulation and use of medications. The first pharmacogenetic testing device, the Roche AmpliChip, was approved by the FDA in 2004 (it assesses genetic markers linked to the function of CYP2D6 and CYP2C19 drug metabolizing enzymes). To date (June 2014), the FDA has relabelled over 140 approved drugs to include genetic information^2^. Among these drugs, 25% are metabolized by cytochrome CYP2D6 and their rates of metabolism can vary; for example, one meta-analysis demonstrated a reduction in 50% in the average dose for most tricyclic antidepressants in patients who are CYP2D6 poor metabolizers (CYP2D6 *3/*3) [16]. Figure 2 shows the distribution of these drugs between various target diseases, with oncology and psychiatry dominating. However, these drug labels do not always provide, based on relevant genetic information, specific guidelines (e.g. in relation to putative adverse drug reactions) and recommendations about what actions should be taken [17].

The most challenging and perhaps most crucial part of such an approach would be the accurate target enrichment of those ADMET-related pharmacogenes, followed by whole-pharmacogenome resequencing. Such an approach would be dynamic in the sense that it would allow enrichment and/or modification of the pharmacogene panel. By contrast, the main disadvantage would be omitting important variants in modifier genes involved in drug metabolism. However, it would definitely capture novel and putative deleterious variants in the known pharmacogenes.

The Pharmacogenomics Research Network (PGRN; http://pgrn.org) and the eMERGE consortium (http://emerge.mc.vanderbilt.edu/) are developing just such a sequence platform targeted to 84 of the most significant pharmacogenes in patients that would be accessible through the eMERGE Network's clinical setting at Mount Sinai Hospital (http://icahn.mssm.edu/research/institutes/institute-for-personalized-medicine/for-researchers-and-innovators/nhgri-emerge-consortium/pharmacogenomics-sequencing-pilot-project). This pharmacogene panel, also referred to as ‘PGRN-seq’ [18], covers variants for so-called tier-1 ‘actionable’ variants/drug–gene pairs, including clopidogrel*/CYP2C19*, warfarin/*CYP2C9–VKORC1* and simvastatin/*SLCO1B1*. The results will be validated against tier-1 other genotyping assays.

For such an approach to be viable and readily applicable in clinical practice, it should be accompanied by the necessary genome informatics platforms to potentiate accurate analysis of the resulting pharmacogenome resequencing, to address the secure storage of the sheer amount of genomic information resulting from pharmacogenome resequencing, and ideally to provide a meaningful and clinician-friendly report so that it can be readily exploited in the clinic. To cope with this challenge, innovative approaches that derive meaningful insights and knowledge from large and complex PGx resources need to be developed and thoroughly tested. The scope of the task is twofold: firstly, to facilitate and enhance identification and evidence-based documentation of (existing or newly discovered) PGx gene/variant–drug–phenotype associations and secondly, the translation and transfer of well-documented PGx knowledge to clinical implementation with the aim of both rationalizing and individualizing the therapy. The following section focuses on this particular challenge.

## 5. Towards pharmacogenomic data integration

As previously mentioned, a crucial component of personalized medicine is the individualization of drug therapy. Understanding the complex interactions and detailed characterization of the functional variants of individual ADMET-related genes and drugs is needed to demonstrate clinical utility. In other words, associating gene variants with specific drug responses in individual patients improves clinical decision-making by informing the adjustment of the dosage or the selection of a different drug [19]. True individualization of therapy, however, which would maximize drug efficacy and minimize toxicity, would need to consider genetic and phenotypic data, as well as any environmental factors that could influence the response to treatment, in the context of the specific individual concerned. Therefore, the design and development of information systems that are continually updatable by inclusion of newly generated pharmacogenomic information, that are able to disseminate knowledge in the form of guidelines, and that are capable of linking the results of pharmacogenomic tests to recommendations for therapeutic interventions with the aim of supporting drug-prescribing decision-makers is a prerequisite for incorporating PGx into routine clinical practice.

Once the Internet became an indispensable tool for biomedical researchers, genomic information overload was inevitable. There are numerous websites and biological databases, which often create confusion for users in terms of which might be the most appropriate to investigate a given biological question. The current battery of genome databases, particularly those directly related to PGx, is limited in number. Table 1 summarizes the resources directly (knowledge bases and genetic testing) or indirectly (databases about gene variants, genotype-to-phenotype associations and drug/chemo-related databanks) related to PGx.

**Table 1.** Web-based resources directly or indirectly related to pharmacogenomics (see also [20,21]).

resource name	description (data and services offered)
PGx knowledge bases	
PharmGKB	The Pharmacogenomics Knowledge Base (www.pharmgkb.org) is a PGx resource that attempts to curate our knowledge of the impact of genetic variation on drug response. It includes clinical information such as dosing guidelines and drug labels, potentially clinically actionable gene–drug associations and genotype–phenotype relationships
CPIC	The CPIC (www.pharmgkb.org/page/cpic) provides peer-reviewed guidelines, in partnership with the journal *Clinical Pharmacology and Therapeutics* (www.nature.com/clpt) and with simultaneous posting to PharmGKB; CPIC guidelines are designed to help clinicians understand how available genetic test results can best be used to optimize drug therapy. CPIC also provides and maintains a list of gene–drug pairs (www.pharmgkb.org/page/cpicGeneDrugPairs)
PGRN	The Pharmacogenomics Research Network (http://pgrn.org/display/pgrnwebsite/PGRN+Home) is a network of scientific groups that is focused on understanding how an individual's genes affect his or her response to medicines; it has been supported since 2000 by the US National Institutes of Health (NIH) to promote discovery and translational research in genomics, in order to enable safer and more effective drug therapies. It leads the TPP initiative with the goal to ‘operationalize the work of CPIC by translating widely accepted actionable pharmacogenetics discoveries into real-world clinical practice’ and to create translational ‘look-up’ tables (www.pharmgkb.org/page/tppTables)
HapMap	HapMap (http://hapmap.ncbi.nlm.nih.gov/) represents an international partnership of scientists and funding agencies from Canada, China, Japan, Nigeria, the United Kingdom and the United States whose goal it has been to develop a haplotype map of the human genome (identifying chromosomal regions harbouring sets of strongly associated SNPs as well as those regions where associations between SNPs are weak). It offers a public resource that helps researchers to find genes associated with human genetic disease and response to pharmaceuticals
CYP Allele Nomenclature	The Human Cytochrome P450 (*CYP*) Allele Nomenclature Database (www.cypalleles.ki.se) provides information on major cytochrome P450s and their genetic polymorphisms, offering gene–allele—enzyme activity tables with literature links
SuperCYP	SuperCYP (http://bioinformatics.charite.de/supercyp/) is a cytochrome P450 database that contains information on about 1170 drugs, 2785 cytochrome–drug interactions and about 1200 alleles in 48 *CYP* genes; it offers searches for: (i) ‘drugs’ to find information on their metabolism, their relationship to involved CYPs, their WHO classification (‘ATC tree’); and ‘drug–drug interactions’ that allows users to enter the names of several different drugs and to check interactions between these drugs and (ii) ‘CYP–drug interactions’ and ‘Polymorphisms’ with all known alleles accompanied by information about their activity or expression (whether decreased or increased)
P450 drug interaction table	This table, maintained by the Department of Medicine at Indiana University (http://medicine.iupui.edu/clinpharm/ddis/main-table/), contains lists of drugs related (based on published evidence) to specific cytochrome P450 isoforms
UGT-allele database	UGT-allele database (http://www.pharmacogenomics.pha.ulaval.ca/cms/ugt_alleles/) offers information and nomenclature on UDP-glucuronosyltransferases with information about *UGT1A* and *UGT2B* SNPs, haplotypes and alleles
PMT database	The University of California, San Francisco Pharmacogenetics of Membrane Transporters (PMT) database (http://pharmacogenetics.ucsf.edu/) provides information on genetic variants in membrane transporter genes; positions of the SNPs and allele frequencies in major racial and ethnic populations are provided; variants are mapped to the gene structure, while variants that alter the protein sequences of the transporters are mapped to the secondary structure of the transporters. Links to information on each transporter in NCBI databases are provided
NAT Gene Nomenclature	The NAT (N-Acetyltransferase) gene nomenclature website was launched to provide an update on known NAT alleles (http://nat.mbg.duth.gr/). New allele submissions are sent to the NAT Nomenclature Committee for review, and the website covers alleles of the *NAT1* and *NAT2* genes. The NAT allele nomenclature follows the star-allele encoding schema with special emphasis on functionally relevant sequence variations
PharmaADME	PharmaADME (www.pharmaadme.org) is an industry-initiated effort that aims to develop a consensus ‘Core List’ of standardized evidence-based drug absorption, distribution, metabolism and excretion (ADME) genetic biomarkers; the ‘Core List’ currently includes 32 ADME genes and 184 markers
e-PKgene	The e-PKGene pharmacogenetics database (http://www.druginteractioninfo.org/applications/pharmacogenetics-database) is a manually curated repository that provides access to available quantitative information on drug exposure contained in the PGx literature; it provides in-depth analysis of the impact of genetic variants of enzymes and transporters on pharmacokinetic responses to drugs and drug metabolites
PGx genetic testing	
FDA PGx Markers	A table of FDA-approved drugs with pharmacogenomic information in their labelling is provided (http://www.fda.gov/drugs/scienceresearch/researchareas/pharmacogenetics/ucm083378.htm). The labelling for some, but not all, of the products includes specific actions to be taken based on the biomarker information
GTR	The Genetic Testing Registry (www.ncbi.nlm.nih.gov/gtr) provides a central location for the voluntary submission of genetic test information by providers; its scope includes the test's purpose, methodology, validity, evidence of the test's utility and laboratory contacts and credentials
Warfarin Dosing	WarfarinDosing (http://www.warfarindosing.org) is an open-access Web site designed to help clinicians initiate warfarin therapy by estimating the therapeutic dose in patients new to warfarin. This site is supported by the Barnes-Jewish Hospital at Washington University Medical Center, the NIH and donations. Estimates are based on clinical factors and (when available) genotypes of two genes: *CYP2C9* and *VKORC1*; recommendations are based on data obtained from over 1000 patients
Variance and G2P databases	
dbSNP	dbSNP (www.ncbi.nlm.nih.gov/projects/SNP) is a public-domain archive containing a broad collection (over 30 million) of simple genetic polymorphisms, SNPs; it is part of the National Center for Biotechnology Information (NCBI; www.ncbi.nlm.nih.gov) and presents the most comprehensive and widely accessed database of human DNA polymorphisms
dbGaP	dbGaP (www.ncbi.nlm.nih.gov/gap) is a public-domain archive that distributes the results of studies that have investigated the interaction of genotype and phenotype; it contains genotypes, pedigree information, fine mapping results and resequencing traces from over 2000 clinical datasets. Searches of dbGaP can be made by disease, genotyping platform or study name, or studies can be browsed
dbVar	dbVar (www.ncbi.nlm.nih.gov/dbvar) is a public-domain database of genomic structural variation that contains data and analyses from studies on large-scale genomic variation and provides associations of defined variants with phenotype information, plus additional documentation including an overview of structural variation; dbVar is linked to ClinVar
ClinVar	ClinVar (www.ncbi.nlm.nih.gov/clinvar) is a public-domain archive of reports of relationships between medically important variants and phenotypes; the database is tightly coupled with dbSNP and dbVar; it is based on the phenotypic descriptions maintained in MedGen (www.ncbi.nlm.nih.gov/medgen)
DGVa	The Database of Genomic Variants (DGVa; www.ebi.ac.uk/dgva) is a database of genomic variants that catalogues, stores and freely disseminates copy number variants in multiple species, providing a valuable resource to a large community of researchers
HGVS	The Human Genome Variation Society (www.hgvs.org) fosters the discovery and characterization of genomic variations including population distribution and phenotypic associations. The Society is an Affiliate of the International Federation of Human Genetics Societies (www.ifhgs.org/) as well as the Human Genome Organization (www.hugo-international.org/)
NHGRI/GWAS	The National Human Genome Research Institute (NHGRI; www.genome.gov) maintains a catalogue of GWAS; the catalogue provides a publicly available manually curated collection of published GWAS assaying at least 100 000 SNPs and all SNP–trait associations with *p* < 10^−5^; it includes 1751 curated publications of 11 912 SNPs. In addition to the SNP–trait association data, the catalogue also publishes a quarterly diagram of all SNP–trait associations mapped to the SNPs' chromosomal locations
PheGenI	The Phenotype–Genotype Integrator (http://www.ncbi.nlm.nih.gov/gap/phegeni) merges data from the NHGRI/GWAS catalogue with several NCBI databases (dbGaP, OMIM, GTEx and dbSNP); it is intended for clinicians and epidemiologists and can facilitate the prioritization of variants for follow-up, study design considerations and generation of biological hypotheses. Users can perform searches based on chromosomal location, gene, SNP or phenotype, and view and download results including annotated tables of SNPs, genes and association results, a dynamic genomic sequence viewer and gene expression data
FINDbase	The Frequency of Inherited Disorders worldwide database (http://www.findbase.org) provides a ‘one-stop shop’ solution for pharmacogenomic marker allele frequency information in over 100 populations and ethnic groups worldwide
PGMD	The PharmacoGenomic Mutation Database (PGMD; http://www.biobase-international.com/product/pgmd) is a resource for identifying all published genetic variants that have been shown to affect drug response in patients. Scientific literature is assembled from literature mining for every *in vivo* patient study that has yielded a significant correlation between genotype and drug response. This database offers multiple delivery models for accessing these data, including an intuitive exploratory interface and a data download for integration with in-house analysis pipelines
Drug/Chemo databases	
KEGG DRUG	KEGG Drug (http://www.genome.jp/kegg/drug) is a comprehensive drug information resource for approved (in Japan, USA and Europe) drugs. It includes information about generic names (associated with chemical structures); representative trade names; links to FDA-approved drug labels information (from DailyMed—a resource which provides high-quality information about marketed drugs; http://dailymed.nlm.nih.gov); chemical structure, chemical component, peptide/polyketide sequence; text description of activity and efficacy; therapeutic category, ATC code and other comments; target molecules in the context of KEGG pathway maps; drug metabolizing enzymes and transporters; other interacting molecules including genomic biomarkers, CYP inducers/inhibitors, etc.; adverse drug–drug interaction data; history of drug development; drug classification information in BRITE hierarchy; links to outside databases
HTD	Human Transporter Database (HTD) (http://htd.cbi.pku.edu.cn) provides a well-organized interface to allow research communities to search detailed molecular and genetic information of drug transporters for the development of personalized medicine. This resource documents 1555 human non-redundant transporter genes, including extensive annotations and global properties of the transporter genes, such as expression patterns and polymorphisms in relationships with their ligands
DrugBank	DrugBank (http://www.drugbank.ca) is a database of drug and drug target information. Apart from extensive data collection on the nomenclature, ontology, chemistry, structure, function, action, pharmacology, pharmacokinetics, metabolism and pharmaceutical properties of drugs, it also includes, as part of its last update, drug-action pathways, drug transporter/metabolite, pharmacogenomic, adverse drug response, ADMET and pharmacokinetic data, making the database much more useful for a wide range of ‘omics’ applications
HGMD	The Human Gene Mutation Database (HGMD; http://www.hgmd.org) collates known (published) gene lesions responsible for human inherited disease. It includes the first example of all mutations causing or associated with human inherited disease, plus disease-associated/functional polymorphisms reported in the literature. HGMD currently lists more than 150 000 variants in more than 6100 different genes including many pharmacogenes

The main problem regarding the exploitation of PGx knowledge and its utilization in clinical practice relates to the heterogeneity and low degree of connectivity between different PGx resources. In most cases, the amount of raw data is so overwhelming that PGx biomedical researchers and stakeholders are often at a loss to know how to make sense of it, rendering them unable to capture all that is known and being discovered regarding genetic variation and its correlation with variable drug response [22]. The challenge to design integrated Web information systems to interconnect and federate diverse PGx information resources into a single portal is a formidable one [23].

## 6. Integrated pharmacogenomic assistant services

The PGx information overload challenge calls for specialized informatics services for the interpretation and integration of the increasingly large amounts of molecular and clinical data. Such a multifaceted endeavour entails both translational and clinical bioinformatics approaches that would, on the one hand, offer analytical and interpretational methods to optimize the transformation of increasingly voluminous biomedical data into proactive, predictive, preventive and participatory (‘4Ps’) medicine [24], and on the other, enable the clinical application of discovery-driven bioinformatics methods to understand molecular mechanisms and prompt the search for potential therapies for human diseases [25].

In this respect, the R&D agenda aims to create and deliver an electronic PGx assistant platform to act as the PGx *bench-to-bedside* enabling medium. The fundamental components that together underpin the novelty of such a platform revolve around its ability to provide translation services which will in turn link genotypic to phenotypic (metabolizer status) information as a valuable tool both to clinicians, by supporting them in making informed decisions based on state of the art PGx data, and to biomedical researchers, by providing a single place where information can be found to facilitate an understanding of inter-individual differences in drug efficacy, toxicity and pharmacokinetics (PK), as well as driving the discovery of new PGx variants.

In this respect, the goal is to provide a ‘one-stop shop’ Web-based platform to ease the processing, assimilation and sharing of PGx knowledge and facilitate the aggregation of different PGx stakeholders' perspectives. The platform should take advantage of, and be designed around, interoperable and flexible bioinformatics and advanced information processing components that are able to serve two major PGx tasks: (i) to offer personalized diagnostics based on reliable genomic/genetic evidence and (ii) to reduce healthcare costs by increasing drug efficacy and minimizing adverse drug reactions.

To develop such a system, one would first need to determine its functional requirements, from the user's perspective. Such requirements would include: (i) retrieval of PGx information regarding ADMET genes, their respective variants and drugs; (ii) a format that is readily updatable with information on newly discovered pharmacogenomic variants; and (iii) the capability to receive personalized recommendations based on personalized PGx profiles. These would impose the main requirements for electronic PGx assistant. However, more detailed requests seem to relate to specific users' roles. In particular, four different types of potential users may be identified in terms of their likely needs and roles: (i) *the individual/patient*: any user who provides single nucleotide polymorphism (SNP) genotype profiles with the aim of receiving corresponding clinical annotations and personalized PGx recommendations (as assessed and validated by healthcare professionals); (ii) *the medical professional*: any healthcare professional (physician, geneticist, etc.) who needs to infer the phenotypic status of individuals (based on their genotype profiles, and by reference to ‘look-up’ genotype–phenotype translational tables), to review and supervise an individual patient's personalized recommendations, assess them and decide upon ensuing therapeutic protocols and treatment options; (iii) *the submitter*: any biomedical researcher who discovers and identifies a new gene variant and its putative PGx associations—the submitter can either validate the findings and enrich the system's database, or request a (local) version of the PGx database to work with; and (iv) *the administrator*: any user with administrative privileges responsible for maintaining and upgrading the electronic PGx assistant's database server (backups, versioning, restoration, etc.), managing application tools and services, assigning and authorizing user roles and privileges, and providing appropriate security and privacy-preserving services.

### 6.1. Towards an electronic PGx assistant

**Figure 3.**
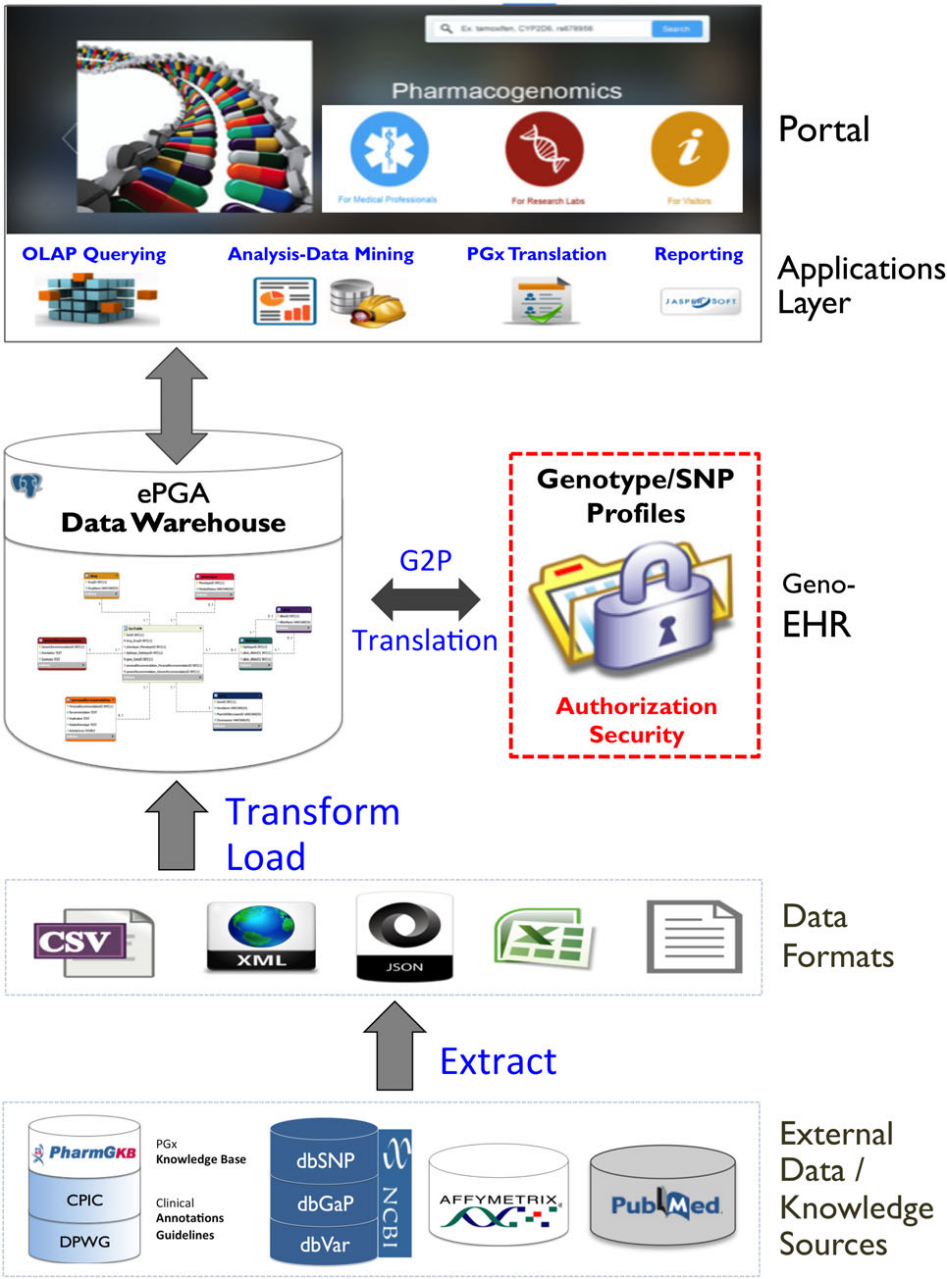
Reference layered architecture of the electronic PGx assistant: components, data/information flow and services.

As a next step, we describe the development of the most crucial components (data model and personal information management) of an electronic PGx assistant, designed on the basis of the aforementioned functional requirements. Figure 3 outlines the reference architecture, in a multi-layer level, including the basic components of the proposed electronic PGx assistant platform.

**Figure 4.**
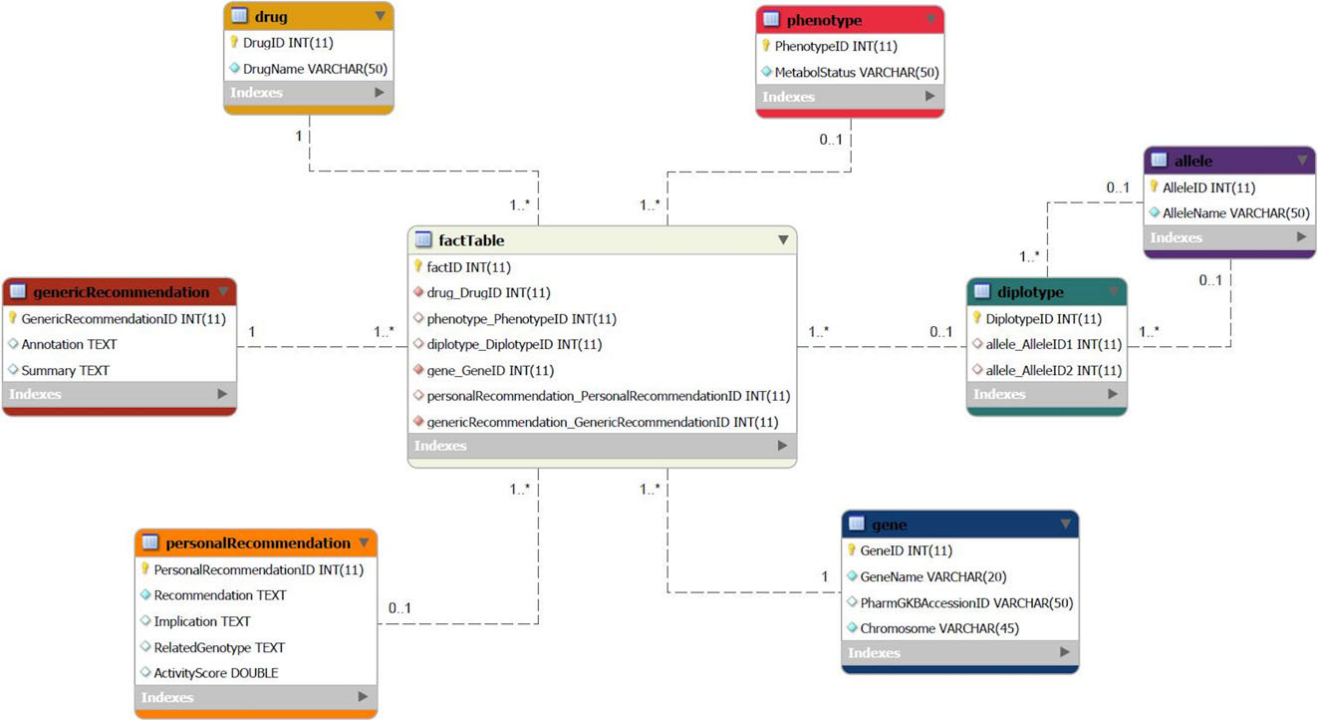
The basic star-schema data model of the electronic PGx assistant: gene—any gene that is known to be associated with drug metabolism; defining attributes: GeneName, Chromosome and PharmGKB code (PharmGKBAccessionID); drug—any PGx drug; defining attributes: DrugName and PharmGKB code (PharmGKBAccessionID); factTable—the table that joins genes, drugs, diplotypes, phenotypes and recommendations; its primary (composite) key is a combination of the foreign keys of other tables; diplotype—the diplotype as defined by the two allele combination (i.e. *1/*2); defining attributes: the two allele IDs (Allele1ID and Allele2ID); allele—any star-allele haplotype (i.e. *1, *2, *1B, etc.); defining attribute: (AlleleName); genericRecommendation—a recommendation for a specific gene–drug combination; defining attributes: Summary and Annotation; personalRecommendation—specific recommendations when diplotype is known; defining attributes: Recommendation, Implication, RelatedGenotype (i.e. an individual carrying two loss-of-function alleles) and ActivityScore; phenotype—the different types of Metabolizer status (i.e. Intermediate Metabolizer, Extensive Metabolizer, etc.); defining attribute: MetabolStatus.

At first, several external data sources are leveraged to extract and integrate pharmacogenomic information. To this end, we adopted the trivial in the design of business intelligence and decision support systems, notion of a Data Warehouse (DW) star schema (figure 4), as the basic data model and the most appropriate to encompass the different requirements for database technology, compared with traditional database systems that support typical online transaction processing applications.

For example, a characteristic question in a medical professional's mind might be: ‘I am about to prescribe aripiprazole to a patient who is an Extensive Metabolizer of this drug. Are there any recommendations?’ or ‘Which drugs are related to the *2/*3A TPMT diplotype?’ The corresponding On Line Analytical Processing Query for the first question would then be (following the proposed DW star schema):

SELECT Recommendation FROM factTable, drug, phenotype WHERE factTable.RecommendationID=Recommendation. RecommendationID AND factTable.DrugID=drug.DrugID AND factTable.PhenotypeID=phenotype.PhenotypeID AND phenotype.MetabolStatus=‘Extensive Metabolizer’ AND drug.DrugName=‘aripiprazole’;

The DW is centred around the gene/variant–drug–phenotype-recommendation concept, embodied in the fact table which, in turn, references the dimensional tables around it, corresponding to the entities of: (i) gene, (ii) drug, (iii) diplotype, (iv) phenotype and (iv) clinical annotations, guidelines and recommendations. These entities correspond to the dimension attributes that act as foreign keys to the fact table. Different types of data extraction tools (e.g. APIs, Web-Services, JSON/XML or text parsers, etc.) are used in order to fetch and transform data from the various heterogeneous data sources (PharmGKB, dbSNP, Affymetrix annotations, PubMed, etc.) into the central DW, following an extraction–transform–load process. Standard ontologies and nomenclatures are used in an effort to uniformly represent the various data and information PGx items (e.g. gene-variant nomenclatures, gene ontology/GO, ICD for disease classification and encoding, etc.).

With regard to the management of individuals' genotype/SNP profiles, an electronic healthcare record (EHR) solution has been adopted. To this end, state-of-the-art guidelines and data-models related to the genetic tests and their interpretations have been employed (e.g. the HL7/CDA2 guide for genetic testing report [26]). Figure 4 depicts an outline architecture for the integration of genotype data from the genetic laboratory to the EHR. Standard ontologies and data-models could be used for the representation of genotype profiles (e.g. genetic variant format, www.sequenceontology.org/resources/gvf.html, and LOINC, https://loinc.org) [27]. The utility of linking genotype data to EHRs is crucial for the translation and transfer of PGx knowledge into the clinic. The approach is both cost-effective and time-efficient as there is no need to actively recruit and gather samples from a study population—cases and controls are readily available and consistently identified from EHRs and the linked genetic samples. The eMERGE (http://emerge.mc.vanderbilt.edu/) consortium has already exploited this alternative with very interesting results [28,29].

Regarding the accumulation, storage and management of individuals' genotype profiles, and taking into consideration the current debate about genetic tests and translational research [30], a number of key ethical issues are raised, viz. public genetic awareness and genomic literacy, physicians' knowledge of genomics, handling of genomic information in and beyond the clinic, online direct-to-consumer (DTC) (pharmaco)genomics, with the associated arguments to be highly polarized [31]. In this respect, all the relevant ethical, privacy preserving and security issues should be employed and implemented, with special effort devoted to surveys and the assessment of guidelines, in order to critically appraise the impact of genetics and PGx on society and increase the level of awareness of the general public, healthcare professionals and biomedical researchers to PGx and personalized medicine.

From the researcher's perspective, the proposed PGx assistant will enhance PGx research by facilitating the discovery of new PGx variants. Methods for discovering genetic factors in drug response, including GWAS, expression analysis and even whole-genome resequencing, already exist. However, more sophisticated knowledge-based tools to assign meaning to novel variants are required. The proposed PGx assistant brings reporting and analytical services to the end user, through a simple and user-friendly interface, and supports research by revealing hidden relations between genes, variants and drugs, thereby driving the discovery of candidate genomic regions of interest. Moreover, as knowledge about drug–gene and drug–drug interactions accumulates, the proposed freely available system, which is additionally coupled with advanced literature mining features and updatable components, becomes even more beneficial to the research community and society.

## 7. Personalized pharmacogenomic translation services

Surely the most important notion in the proposed PGx assistant platform is the idea of personalization. The inclusion of a personalized PGx translation component in the platform is founded on the assumption that clinical high-throughput and pre-emptive genotyping will eventually become common practice and clinicians will increasingly have patients' genotypes available before a prescription is written [32]. The personalized translation component aims to serve: (i) the automated matching of patients' genotype profiles with established and/or newly discovered gene-variants/alleles—based on the customization of an elaborate allele-matching algorithm [33]; (ii) the inference of respective phenotypes (e.g. metabolizer profiles); and (iii) the delivery of relevant and updated clinical annotations and (drug) dosing recommendations. The component will be founded upon the harmonization of PGx haplotype/translation tables from the DMET Plus assay and PharmGKB knowledge base (www.pharmgkb.org), but will also provide services for updating the haplotype/translation tables with newly discovered and validated gene-variants/alleles.

In order to produce accurate personalized recommendations, the focus should be placed on the relationship between a variant and its related gene, drug(s) and phenotype(s). For example, let us suppose that a patient with bipolar disorder receives a genetic test that has the potential to determine an A/C genotype for the variant rs2032582, which corresponds to the 7 : 87160618 nucleotide position of the *ABCB1* gene. *How can this information be translated into clinical knowledge*? The medical professional should be well aware that although the majority (70–80%) of patients with bipolar disorder respond well to lithium, a significant proportion will present with patterns of partial or non-responsiveness. Before prescribing this drug, the physician enters the patient's encrypted genotype data into the proposed PGx assistant, which then identifies that the specific variant is related to lithium response. More specifically, the medical professional receives the following information, that the PGx assistant would bring to his/her attention: ‘Patients with the AC genotype and depression may have an increased risk of suicidal ideation when treated with clomipramine, lithium,…, or venlafaxine as compared to patients with the CC genotype’ (relevant text from PharmGKB). At the same time, the PGx assistant displays three new studies related to genetic variants associated with lithium response and provides links to the respective sources of information. By considering the patient's family and medical history, the medical professional may then decide to provide an alternative treatment and closely monitor this patient. The medical professional may also prescribe a new genetic test based on the findings of the recommended articles in the literature that associate additional genetic variants with response to lithium.

A related protocol, called PG4KDS, is employed at St Jude Children's Research Hospital (http://www.stjude.org/pg4kds). The purpose of the protocol is to selectively migrate microarray-based genotypes for clinically relevant genes into each patient's electronic medical record pre-emptively. By leveraging ‘look up’ translation tables created by the Translational Pharmacogenetics Project (TPP) [34], a PGRN-led initiative with the goal of operationalizing the work of the Clinical Pharmacogenetics Implementation Consortium (CPIC) [35] by translating widely accepted actionable PGx discoveries into real-world clinical practice, they assigned phenotypes to each unique *CYP2D6* or *TPMT* diplotype based on assessments of functional allele activity [36].

## 8. Encouraging pharmacogenomic data sharing

The advances in bioinformatics required to annotate human genomic variants and to place them in public data repositories have not kept pace with data discovery. The continued deposition of such data in the public domain is essential to maximize both their scientific and clinical utility. However, rewards for data sharing are few, representing a serious practical impediment to data submission, and as such, incentivizing individual researchers or research groups to submit their newly acquired and unpublished mutation/variation data to public repositories or knowledge bases in return for appropriate credit, and attribution is of the utmost importance.

In 2008, the scientific journal *Nature Genetics* introduced the concept of ‘microattribution’, to introduce an alternative reward system for scientific data contributions. The principle of microattribution is ‘… to produce a publication workflow that is open to all journals and that draws on the expertise of all those with a stake in understanding variation at a particular region in the human genome’ [37]. Microattribution comprises two main components, namely the Public Genome Browser, to display the actual number of database entries and related articles that would be contributed and recorded, based on an individual researcher's unique identity (e.g. Open Researcher and Contributor ID consortium, http://orcid.org; ResearcherID, http://www.researcherid.com, etc.), and the microattribution analysis article, which would summarize the features of all variome data contributions, such as phenotypes, clinical findings, allele frequencies and so on [38].

The first demonstration of microattribution working in practice was achieved by Giardine *et al*. [39] using HbVar, the globin-gene locus-specific mutation database, as a model, followed by the InSiGHT locus-specific database much later [40]. This approach has also been implemented in the field of clinical genetics [41], documenting the clinical features of almost 40 000 cystic fibrosis patients and their underlying *CFTR* gene variants. Lastly, microattribution has also been implemented in the FINDbase database (http://www.findbase.org), a worldwide national genetic database documenting causative mutations and pharmacogenomic biomarkers [42], followed by the Pharmacogenomics for Every Nation Initiative consortium (http://www.pgeni.org [43]).

In all of the above cases, not only were the overall contributions from individual scientists increased compared with the situation prior to the microattribution call, but most importantly, a number of useful conclusions were drawn in every case that the microattribution was implemented, derived from, for example, variant clustering, clinical phenotype and/or pharmacogenomic variant allele frequencies comparisons and so on. Such conclusions would not have been possible without such an approach, further demonstrating the value of the immediate contribution and sharing of novel genome variants even though they would not warrant classical narrative publication on their own.

In the context of human PGx variation data sharing, a sensible approach to incentivize free data sharing would be to base the whole process around one or more pre-existing and freely available high-quality centralized databases or database journals, possibly coupled with the regular publication of microattribution-type articles (possibly online only) in PGx journals, so that the individual contribution of the data submitters in a consortium would be recognized by their co-authorship. Such an approach would in turn further stimulate researchers to submit data to a central repository.

A significant hurdle that needs to be overcome is the self-sustainability of such a large centralized database, possibly by partnering with a major publishing group, along the lines of a database-journal-like model [38]. One should also bear in mind that the variation data to be generated from such pharmacogene resequencing would be ‘raw’ uncurated data. Being different from well-curated data, they would have to be handled differently, e.g. tagged as uncurated data, since for instance, they might correspond to benign rather than pharmacogene-disabling variants (see also [10]), and specify the level of data confidence in a clear way, e.g. raw (and perhaps false positive) data versus curated data of unknown significance or with an *in silico* prediction or even with a clear genotype–phenotype correlation.

## 9. Translating PGx knowledge into clinical decision-making: the next great leap

From the above, it is clear that once next-generation re-sequencing-based PGx testing becomes widely available, it will require a substantial effort to translate this genomic information into clinically meaningful guidelines. In the real life situation, the PGx clinical scenarios are truly complex, which often, if not always, poses significant dilemmas to the medical professionals regarding the selection of a treatment modality. This complexity does not occur because of our inability to correlate genomic with clinical variables; indeed, genomics research has already revealed and produced (and continues to produce) a plethora of valuable pharmacogenomic associations and knowledge. This complexity arises mainly due to the large *translation gap* in moving pharmacogenomic (as with the other—omics) scientific discoveries towards successful innovations. This gap occurs because of the lack of a ‘systems orientation’ to innovation that conceptualizes knowledge-based PGx innovation as an ecosystem of communicating ‘innovation actors’ (pharmacology, pharmacogenomics, molecular biology and genetics researchers) and ‘innovation narrators’ [44], an ecosystem to be realized by the (currently) missing ‘intermediate medium’ that facilitates communication and supports *bench-to-bedside* translation endeavours, by harnessing knowledge from basic PGx to produce treatment options for patients [45].

In such a setting, it is imperative to adopt a multidisciplinary approach based on a portfolio of interoperating translational or clinical biomedical informatics components and their alignment with contemporary information engineering and processing approaches. Such approaches should aim to devise: (i) a PGx knowledge assimilator that seamlessly (i.e. based on standard semantics and data-models) links diverse PGx knowledge sources and (ii) knowledge-extraction services able to identify useful genotype-to-phenotype associations and knowledge from these sources. Moreover, the identified PGx genotype-to-phenotype associations should be explored in relation to their PK and pharmacodynamic (PD) background. Such exploration could be served by the elaboration of the appropriate PK/PD simulation models that help to assess PGx association's covariance in virtually devised populations, e.g. following the approach of SimCYP (www.simcyp.com/) and NONMEM (www.iconplc.com/technology/products/nonmem) virtual simulation commercial packages, as well as using free open-source PK modelling s/w tools such as ‘PKreport’ (cran.r-project.org/web/packages/PKreport) and ‘WFN’ (wfn.sourceforge.net/wfnxpose.htm) R-packages. In addition, and based on the coupling of Web 2.0 and social-networking technology, it would be essential to facilitate and support the engaged collaboration needs and ‘fill-in’ the missing communication medium between the diverse PGx knowledge sources, the simulated PGx genotype-to-phenotype associations and the PGx actors.

To accommodate these needs, one should incorporate: (i) the linkage and seamless integration of established PGx resources (e.g. PharmGKB, CPIC, etc.), literature and other genomic databases (PubMed, dbSNP, dbGAP, ClinVar, FINDbase, etc.), to be based on the elaboration and operationalization of standard (pharmaco)genomic/clinical ontologies and data-models; (ii) literature mining/natural language processing, to extract putative disease–drug–gene/variant–phenotype associations from PGx resources and the published literature; (iii) a virtual population pharmacokinetic simulator, to test putative variant–phenotype associations and assess relevant genotype-to-phenotype covariance statistics in virtual populations; and (iv) a collaborative recommender environment, to enable communication and collaboration between PGx actors towards the formation, validation and evidential assessment of such associations. In addition, two additional components and respective services are required to align and harmonize such a platform with a bench-to-bedside orientation and its utilization in a clinical decision-making setting. First, an Electronic Healthcare Genotype component, that would be readily compatible with the general EHR, so as to service the management of patients' genotype profiles, and a Phenotype-to-Genotype-translation component, to service the automated matching of patients' genotype profiles with established and/or newly discovered gene/variant alleles, inference of the respective phenotypes (e.g. metabolizer profiles), and delivery of up-to-date relevant clinical annotations and respective (drug) dosing guidelines. Finally, a portal will be required as a single-access-point PGx environment that embraces the aforementioned components and services.

Such a system, once operable, would facilitate the integration and translation of PGx knowledge into the clinical decision-making process and bring clinic-based genomic medicine closer to a reality. To this end, one would also need to circumvent additional fundamental hurdles, namely (i) ensuring that all the necessary consents are provided by the patients, (ii) safeguarding sensitive personal data to avoid the inappropriate leaking of genetic information which may lead to stigmatization and (iii) enhancing the genetics awareness and genetics education of healthcare professionals. Related to these issues is the increase in ‘DTC’ genetic testing which, quite apart from its controversial status, has gained a lot of attention in recent years and has already produced interesting results [46]. As these topics lie outside the scope of this article and have been discussed elsewhere [47], they will not be further discussed here.

## 10. Conclusion and future perspectives

The post-genomic revolution, characterized by the rise of massively parallel whole-genome and exome sequencing, has led to the correlation of specific genomic variants with disease predisposition and other clinical features, including response to some of the most commonly prescribed drugs. As personalized drug treatment and genomic medicine gets closer to becoming a reality, the use of whole-genome sequencing that spans all ethnicities and covers all possible genetic alterations is the most useful approach [48]. Recent evidence, though limited at the present time, confirms that whole-genome sequencing can reveal a relatively large number of unique (or rare) pharmacogenomic markers that would otherwise go undetected by conventional genetic screening methods.

An important aspect of the next-generation sequencing technology that would be critical for its early adoption in the clinic is its cost-effectiveness. In other words, it becomes clear that performing a comprehensive personalized pharmacogenomic profile using whole-genome sequencing (currently 3000 US$ and decreasing), that would include almost all of the germline and de novo genomic variants needed to manage all current and future treatment modalities, would be cost-effective when compared with the cost of testing for a single marker or several markers in a few pharmacogenes (from 300 US$ up to 1500 US$, respectively). At present, setting up a (centralized) whole-genome sequencing facility and pharmacogenomic data translation to clinicians are two of the most important hurdles to be overcome, but sample outsourcing for data analysis and interpretation might be the answer to surmounting this obstacle using an economy-of-scale model. Ultimately, as pharmacogenomic testing costs using whole-genome sequencing and cost-effectiveness are well documented, it should only be a matter of time until the cost of pharmacogenomic testing reimbursement is adopted by national insurance bodies.

In light of the above, the design and development of advanced informatics solutions that ease to fill-in the gap between PGx research findings and clinical practice emerges as a major need. Here, we presented the operational requirements and design specifications of an electronic PGx assistant that aims to act as the medium between the various PGx communities (biomedical researchers, geneticists, healthcare providers and PGx regulatory bodies), equipping them with innovative services that enable PGx research findings to reach clinical implementation. The orchestration of the provisioned PGx assistant's services in an integrated platform empowers the capabilities of PGx communities to grasp, assess and maximize the use of relevant biomedical and molecular PGx knowledge. Finally, the implementation of PGx assistant services should address and provide feasible solutions to challenges related to the PGx annotation of whole genomes [49] that concern: the accuracy of PGx markers across the genome; the ambiguity of gene-variants and PGx markers in relevant literature references; the effect of multi gene-variants and PGx markers on individual phenotypes; the combined effects of variants on multiple drugs; as well as the limited body of clear guidelines and recommendations.

## Endnotes

1 These terms are often used interchangeably. However, *pharmacogenomics* refers to the collective impact that many individuals' genetic variations have on the response to medication; by contrast, *pharmacogenetics* refers to the impact of one specific variation, or the impact of variation in one specific gene, on the response to medication.

2 ‘Table of Pharmacogenomic Biomarkers in Drug Labels’; www.fda.gov/drugs/scienceresearch/researchareas/pharmacogenetics/ucm083378.htm.
